# Cortical Effects of Noisy Galvanic Vestibular Stimulation Using Functional Near-Infrared Spectroscopy

**DOI:** 10.3390/s21041476

**Published:** 2021-02-20

**Authors:** Bulmaro A. Valdés, Kim Lajoie, Daniel S. Marigold, Carlo Menon

**Affiliations:** 1 Menrva Research Group, Schools of Mechatronic Systems and Engineering Science, Simon Fraser University, 250-13450 102nd Avenue, Surrey, BC V5A 1S6, Canada; bulmaro.valdes@gmail.com (B.A.V.); kim_lajoie@sfu.ca (K.L.); 2 Sensorimotor Neuroscience Lab, Department of Biomedical Physiology and Kinesiology, Simon Fraser University, 8888 University Drive, Burnaby, BC V5A 1S6, Canada; daniel_marigold@sfu.ca; 3 Biomedical and Mobile Health Technology Laboratory, Department of Health Sciences and Technology, ETH Zurich, 8008 Zurich, Switzerland

**Keywords:** functional near-infrared spectroscopy, noisy galvanic vestibular stimulation, non-invasive brain stimulation, stochastic stimulation

## Abstract

Noisy galvanic vestibular stimulation (nGVS) can improve different motor, sensory, and cognitive behaviors. However, it is unclear how this stimulation affects brain activity to facilitate these improvements. Functional near-infrared spectroscopy (fNIRS) is inexpensive, portable, and less prone to motion artifacts than other neuroimaging technology. Thus, fNIRS has the potential to provide insight into how nGVS affects cortical activity during a variety of natural behaviors. Here we sought to: (1) determine if fNIRS can detect cortical changes in oxygenated (HbO) and deoxygenated (HbR) hemoglobin with application of subthreshold nGVS, and (2) determine how subthreshold nGVS affects this fNIRS-derived hemodynamic response. A total of twelve healthy participants received nGVS and sham stimulation during a seated, resting-state paradigm. To determine whether nGVS altered activity in select cortical regions of interest (BA40, BA39), we compared differences between nGVS and sham HbO and HbR concentrations. We found a greater HbR response during nGVS compared to sham stimulation in left BA40, a region previously associated with vestibular processing, and with all left hemisphere channels combined (*p* < 0.05). We did not detect differences in HbO responses for any region during nGVS (*p* > 0.05). Our results suggest that fNIRS may be suitable for understanding the cortical effects of nGVS.

## 1. Introduction

Subthreshold noisy galvanic vestibular stimulation (nGVS) is a non-invasive technique that delivers noisy, alternating electrical current through surface electrodes on the mastoid bones. This stimulation activates both vestibular hair cells and afferents of the otoliths and semicircular canals [1,2]. nGVS can improve standing balance [3,4,5], walking [5,6,7,8], upper-limb function [9,10], vestibular-ocular function [11,12], sensory perception [13,14], memory [15], and visuospatial navigation [16]. The improvements in motor function are also evident in clinical populations, such as those with bilateral vestibulopathy [3,17]. The proposed mechanism for the improvements seen in human performance is via the phenomenon of stochastic resonance [13,14,18]. Stochastic resonance refers to the improved ability to detect stimuli, or better output signal quality, in a nonlinear system with the addition of noise [19,20]. The effects of nGVS are likely the result of the fact that the vestibular afferents terminate in the vestibular nuclei, which can influence motor behavior through their connections with oculomotor circuitry and via the vestibulospinal tract [21,22,23]. Since the vestibular nuclei have dense connections with the thalamus, nGVS has the potential to affect a variety of brain regions and thus behavior [24]. However, knowledge of how nGVS affects cortical activity is still limited.

A few studies have used functional magnetic resonance imaging (fMRI) and positron emission tomography (PET) to show where in the brain non-noisy, suprathreshold direct current [25,26] or sinusoidal [27,28,29] galvanic vestibular stimulation influences. This work reported activations in several brain regions, including the parieto-insular vestibular cortex (PIVC) and supramarginal gyrus (BA 40). It is unclear whether subthreshold nGVS activates similar regions.

Functional near-infrared spectroscopy (fNIRS) is a non-invasive technology that uses near-infrared light to measure relative cortical changes in oxygenated (HbO) and deoxygenated (HbR) hemoglobin as a proxy for neural activity [30]. The electrical currents from nGVS equipment do not interfere with the fNIRS optical readings [31], making this a particularly well-suited technology to determine how this stimulation might affect cortical activity. In addition, fNIRS does not suffer from the same limitations as fMRI and other neuroimaging modalities, such as having to restrict lower- and upper-limb movements, and it is safe and considerably more portable and affordable [32]. Since many human behaviors involve movement, it is important to have the ability to monitor how nGVS affects cortical activity during such tasks and is related to functional improvements. The advantages of fNIRS—its portability and affordability—make it also suitable to study how changes in cortical activity induced by nGVS impact different cognitive behaviors as well.

Previous studies using fNIRS to monitor cortical activity in response to vestibular stimulation have only focused on non-noisy stimulation paradigms, such as head/body movements [33,34], caloric vestibular stimulation [35,36], and suprathreshold direct current galvanic vestibular stimulation [37]. Consequently, as an initial step towards the wider use of fNIRS in brain stimulation research, we had two objectives for this study: (1) determine if fNIRS can detect cortical changes in HbO and HbR with the application of subthreshold nGVS, and (2) determine how subthreshold nGVS affects this fNIRS-derived hemodynamic response in a resting-state paradigm.

## 2. Materials and Methods

### 2.1. Participants

A total of twelve healthy right-handed adults (6 females and 6 males, age: 29 ± 6 years) participated in this study. Exclusion criteria included: metallic implants in the head or neck; brain or spinal cord surgery; epilepsy or seizures; allergy to rubbing alcohol and/or conductive paste/gel; uncorrected visual impairment; severe skin condition at the electrode site; musculoskeletal injury or a condition affecting standing or walking; syncope or fainting spells; concussion or head trauma in the last year; electronic implants in the body; pregnancy or possibility of; consumption of recreational drugs, coffee, or alcohol 8 h before the study; neurological, auditory, or vestibular condition. The Office of Research Ethics at Simon Fraser University approved the study and participants gave informed written consent before participating. Data availability statement is included at the end of the manuscript.

### 2.2. Noisy Galvanic Vestibular Stimulation

An isolated current stimulator (A395R, World Precision Instruments, Sarasota, FL, USA) connected to two round electrodes (2 in., UltraStim X, Axelgaard Manufacturing Co., Ltd., Fallbrook, CA, USA) placed on the participants’ mastoid processes provided the stimulation. We cleaned the skin with alcohol before placing the electrodes and used conductive gel (Spectra 360, Parker Laboratories Inc., Fairfield, NJ, USA) to improve connectivity. We also secured the electrodes in place with adhesive tape (Nexcare, 3M, Saint Paul, MN, USA) to prevent peeling and/or displacement.

We used MATLAB (MathWorks, Natick, MA, USA) custom scripts to generate the noisy signal, which was then sent to the stimulator at a rate of 60 Hz via an acquisition card (USB-6002, National Instruments Corporation, Austin, TX, USA). The signal had a 1/f power spectrum with a zero-mean linearly detrended Gaussian distribution in the range of 0.1–10 Hz. Previously, nGVS studies have used these parameters in individuals with and without movement disorders [9,10,38].

At the beginning of the testing session, we measured each participant’s cutaneous threshold by delivering the noisy signal. Current increased in increments of 20 µA from a baseline value of 10 µA, and participants indicated whether they could feel a tingling sensation for 10 consecutive seconds. If they could not, we increased the current by another step. Once participants reported feeling the stimulation, we set the signal to 80% of this current value [5,8,17]. Subsequently, we confirmed that participants did not feel the current (average peak current: 183 +/− 95 µA), which we delivered as the stimulation magnitude for the experimental session.

### 2.3. Functional Near-Infrared Spectroscopy

**Figure 1:**
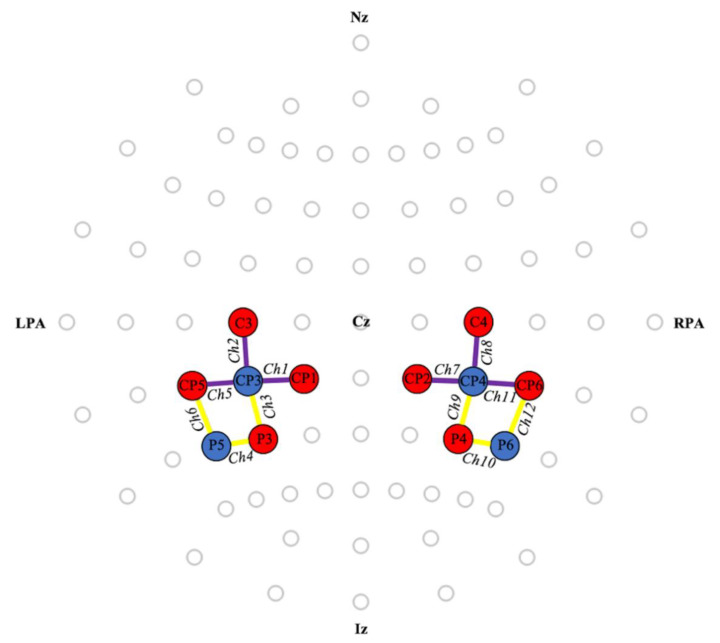
Locations of optodes and channels relative to the 10/10 international system. Detectors and sources are shown in blue and red, respectively. BA40 (**purple**) included channels 1, 2, and 5 on the left hemisphere and 7, 8, and 11 on the right hemisphere. BA39 (**yellow**) included channels 3, 4, and 6 on the left hemisphere and 9, 10, and 12 on the right hemisphere. Ch: Channel. Cz: Vertex. Iz: Inion. LPA: Left pre-auricular. Nz: Nasion. RPA: Right pre-auricular.

We measured relative changes in HbO and HbR using an 8 × 8 NIRSport 2 system (NIRx, Medical Technologies LLC, Berlin, Germany), operating at 760 and 850 nm wavelengths. We used BA40 as the primary region of interest (ROI), as it is involved in vestibular processing [24,39]. BA39 was chosen as the secondary ROI, as it showed activation with the application of GVS in previous fMRI studies [24,29]. We placed optodes on locations with >25% ROI specificity according to the fNIRS Optode’s Location Decider (fOLD) [40]. Figure 1 shows relevant channels and their locations. Aurora 1.3 software (NIRx, Medical Technologies LLC, Berlin, Germany) captured the data at 8.7 Hz.

### 2.4. Experimental Procedure

**Figure 2:**
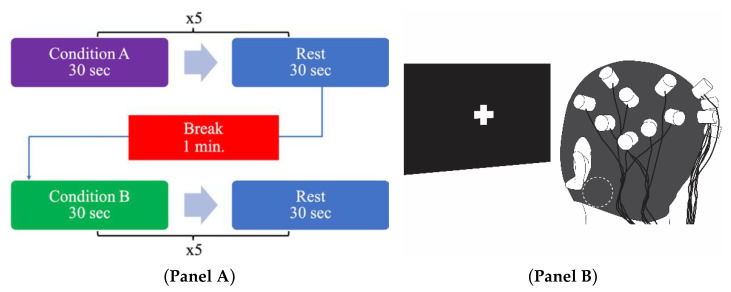
**Panel A:** Experimental design. Participants were randomized to start with nGVS or sham stimulation. After completing 5 repetitions of Condition A and Rest, participants took a 1-min break before completing 5 repetitions of Condition B and Rest. **Panel B:** Experimental setup. Participants wore fNIRS cap and focused on a crosshair while receiving nGVS or sham stimulation. Dotted circle indicates approximate location of stimulating electrode.

We block randomized (block size: 2, pseudo-random number generator [41]) participants to start with either nGVS or sham stimulation (Figure 2, Panel A). The only difference between nGVS and sham stimulation conditions was that, during sham trials, no electrical stimulation was delivered via the electrodes. Participants sat on a standard office chair with locking casters. We instructed them to avoid moving the trunk, head, and jaw as much as possible to prevent motion artifacts. They were blinded to the order of stimulation (Sham vs. nGVS) and told that nGVS would be applied at random intervals. The experiment began once participants expressed that they were ready, approximately 13–50 s after commencing the data recording. During both stimulation conditions, participants focused for thirty seconds on a crosshair displayed on a computer screen in front of them (Figure 2, Panel B). They then rested quietly for another thirty seconds, in which they were free to stop focusing on the crosshair. Repeating trials for specific conditions was similar to experimental designs from previous studies [42,43]. Participants completed five repetitions of the stimulation and rest combination before taking a 1-min break. During the break, they remained seated and silent. After the break, they performed five more repetitions of the remaining stimulation condition and rest combination.

### 2.5. Data and Statistical Analysis

We analyzed data in a similar manner to other studies [42,43,44]. In terms of preprocessing steps, The MATLAB-based Homer 2 toolbox [45] first converted raw fNIRS signals into optical density changes, then into HbO and HbR concentrations. Specifically, we pruned the channels if the raw data had a signal-to-noise ratio < 3. We also used the hybrid spline interpolation and Savitzky-Golay method [46] to correct optical density values for motion and applied a 0.5 Hz low pass filter [47]. We converted optical density signals to HbO and HbR concentrations using the modified Beer–Lambert law with a partial pathlength factor of 6.0 [48]. We calculated the hemodynamic response function with ordinary least squares to solve the general linear model using consecutive Gaussian functions with separations and standard deviations of 0.5 s, and then applied a 3rd order polynomial drift correction [42,44,46]. The time course for the function was from 2 s before the onset of the stimulation to 10 s after the stimulation was turned off.

To investigate whether nGVS elicited cortical effects in the selected ROIs, we compared the differences between nGVS and sham HbO and HbR group concentrations using one-sample t-tests in SPSS Statistics (IBM Corp., Armonk, NY, USA). For each participant, we calculated the HbO and HbR average channel concentrations between 3 and 30 s after the onset of nGVS across the five trial repetitions. We chose this time interval to account for a delay in the hemodynamic response following the onset of the stimulation. Although the exact delay time for this type of stimulation and paradigm is unknown, starting this time window at values between 0 to 5 s after the onset of nGVS produces similar results to those reported below. We then averaged the channels corresponding to each ROI (Figure 1) to obtain a measure for the specific brain regions. Due to the exploratory nature of this study, we also compared the per-channel concentrations to examine which channels the application of nGVS impacted, and we did not correct statistical tests for multiple comparisons.

## 3. Results

**Figure 3:**
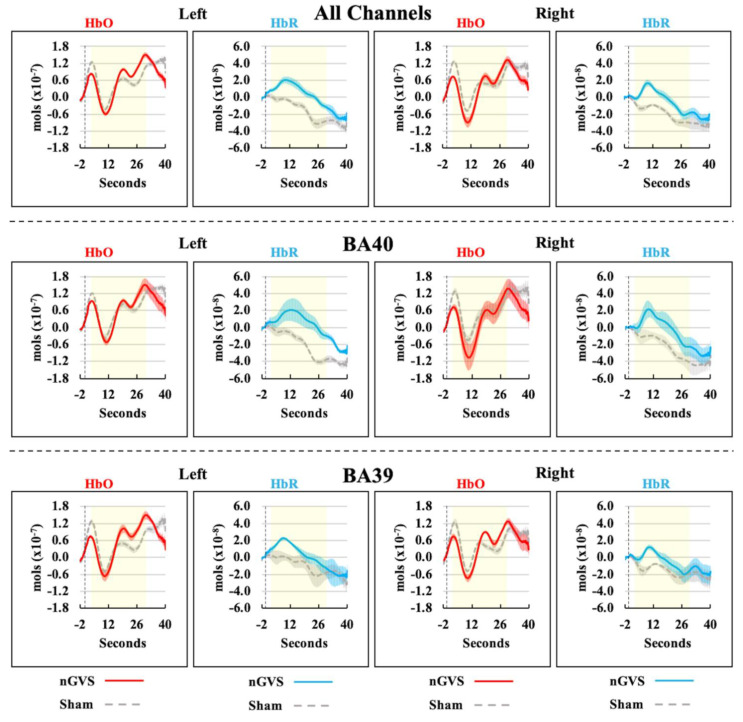
Hemodynamic response function for HbO and HbR for all channels combined (**top panel**), BA40 (**middle panel**), and BA39 (**bottom panel**) in nGVS and sham conditions. Concentration changes (mean: solid line; standard error: shade) of channel combinations vs. time (s) are presented. Columns 1 and 2 correspond to values of the left hemisphere, whereas columns 3 and 4 corresponds to values of the right hemisphere. Vertical dashed lines indicate the onset of nGVS (i.e., time 0). Participants received stimulation from 0 to 30 s. Yellow rectangle indicates analysis window (i.e., 3 to 30 s after stimulation onset).

Figure 3 shows the hemodynamic response functions for HbO and HbR in the nGVS and sham conditions. From visual inspection, mean values for HbO during the nGVS and sham conditions tended to remain above zero, with the response slightly higher in the nGVS condition, particularly in relation to the left hemisphere. For HbR, mean values were elevated in the nGVS condition compared to those in the sham condition for the whole duration of the noisy stimulation. This is evident in all channels as well as BA40 and BA39 analyses.

Table 1 highlights statistical test results for the different ROIs. We did not detect statistically significant differences between nGVS and sham, in either ROI, or their combination when comparing HbO average concentration values during the analysis window. For HbR, values in the nGVS condition for the left BA40 (*p* = 0.032), and for the left hemisphere when all channels were combined (*p* = 0.040), were significantly different from those in the sham condition. For the left hemisphere, 9/12 and 10/12 participants showed an increase in HbR when receiving nGVS when all channels were combined, and when focusing on BA40, respectively. HbR average values on the right hemisphere indicated a trend for BA40 (*p* = 0.079) and when combining all channels (*p* = 0.065). For the right hemisphere, 9/12 participants showed an increase in HbR while receiving nGVS when all channels were combined, and when focusing on BA40.

When comparing per-channel concentration mean values (Table 2) for HbO, none of the channels were significantly different in the nGVS condition compared to sham. For HbR, channels 1 (*p* = 0.035, left hemisphere) and 7 (*p* = 0.047, right hemisphere) were significantly different when participants received nGVS compared to the sham condition. Both channels formed part of BA40 and were on the same location, but opposite hemispheres. Channels 2 (*p* = 0.071, BA40 left hemisphere) and 3 (*p* = 0.073, BA39 left hemisphere) displayed only a trend towards significance.

Only a small sample of participants reported experiencing minor adverse effects related to the vestibular stimulation. This is shown in Table 3.

## 4. Discussion

In this work, we explored whether it is possible to monitor the effects of subthreshold nGVS on cortical brain activity with fNIRS and how HbO and HbR concentrations are affected by this form of vestibular stimulation. The majority of participants in this study showed different cortical responses when they received nGVS versus sham stimulation. In particular, the results showed higher HbR concentration during nGVS stimulation in a region previously associated with vestibular processing.

The increase in HbR with nGVS differs from the typical hemodynamic pattern of increased HbO and decreased HbR responses—thought to reflect increased neural activity—observed in most fNIRS studies [30,32]. However, past fNIRS studies have occasionally reported increased HbR responses, often referred to as an inverse (or negative) oxygenation response, during a variety of tasks, including motor imagery and visual stimulation [49,50,51]. This inverse response relates to the negative blood-oxygenation-level-dependent signals seen with fMRI [50]. What might cause the increased HbR response? Although we cannot definitively address this question within the context of our study, there are several possible explanations that relate to the complex interaction between neuronal activity, cerebral blood flow (CBF), and cerebral metabolic rate of oxygen consumption (CMRO_2_). For instance, increased HbR responses may reflect increased neural activity with an increase in CMRO_2_ and either no change or a decrease in CBF; decreased CBF alone; or reduced neuronal activity leading to a greater decrease in CBF than a decrease in CMRO_2_ [30,49,50,52,53]. Recent work would suggest that the increased HbR response we observed is due to a decrease in neuronal activity [53,54,55], which in turn may relate to inhibitory circuits with, or reduced activity from, the vestibular nuclei and their widespread projections.

We found that nGVS led to a significant increase in HbR concentration in channels likely corresponding to BA40 of the left hemisphere compared to sham stimulation, with no change in HbO concentration. A similar pattern occurred in the right hemisphere, though the HbR concentration did not quite reach statistical significance. Owing to rich vestibulo-thalamo-cortical connections, not surprisingly, fMRI, PET, and MEG studies have shown that caloric and galvanic vestibular stimulation leads to neuronal activation in numerous cortical regions [24]. In fact, these stimulation paradigms have repeatedly shown activation in supramarginal gyrus (BA40) as well as adjacent regions, including angular gyrus (BA39), superior temporal gyrus (BA22), and BA7 [24,25,28,29,35,56,57]. However, our findings are suggestive of deactivation in BA40. Stephan et al. [29] did show blood-oxygen-level-dependent signal decreases (i.e., deactivations) following sinusoidal galvanic vestibular stimulation in and around right BA39 using fMRI. More recently, Becker-Bense et al. [27] reported bilateral deactivations in similar areas, among others, using PET and fMRI. Other studies have also shown deactivations following direct current galvanic vestibular stimulation in a range of brain areas [25]. Although we refer to BA40 in our work, it is not possible to define the precise location of where our signals originate given that fNIRS has less spatial precision than PET and fMRI. Thus, it is highly possible that we are also recording from these adjacent areas [58]. Despite a broad range of cortical areas responding to vestibular stimulation, the nearby parietal insular vestibular cortex (PIVC) is thought to represent the main vestibular processing center [59,60]. Recent work suggests the central human vestibular cortex consists of PIVC and an adjacent area called the posterior insular cortex, together referred to as the PIVC+ complex [59]. These regions are densely connected to the more superficial supramarginal gyrus (BA40) and superior temporal gyrus in humans [61]. Taken together, our results demonstrate the ability to use fNIRS in nGVS studies. Given that cortical activation is also evident with caloric vestibular stimulation and fNIRS [35], fNIRS technology may be useful to better understand cortical vestibular processing, particularly when paired with other neuroimaging modalities.

There are several limitations of our study. First, fNIRS technology is limited in that it measures from more superficial cortical layers. Second, we recorded from a relatively small area of the brain. Although we selected BA40 and BA39 based on past research, it is unclear whether changes in HbO and HbR concentrations with nGVS are present in other cortical areas when using fNIRS. Third, we did not use MRI of each participant to determine optode positioning, though we did use a recognized fNIRS toolbox [40]. Fourth, a variety of physiological signals can affect cerebral hemodynamics and, thus, the attribution of the observed effects to functional brain activity [62]. Although we used signal processing techniques—similar to others—to prepare the data for analyses and to minimize these artefacts from contaminating our data, there remains the possibility that we observed false positives. We did not have the capability to use short-separation channels with our fNIRS system, which could help mitigate these effects [62]. However, future work should include them when possible. Fifth, we used a simple resting-state paradigm. However, we believe this provided necessary control to determine the effects of nGVS with fNIRS before using more complex cognitive or movement-related tasks. This is an important first step toward more widespread use of this technology when applying nGVS. Sixth, our noisy stimulation parameters matched several studies [9,10,38] but differed from many others [4,5,7,8,17]. How different noisy stimulation parameters influence HbO and HbR concentrations is unclear at this time.

Overall, our results support the use of fNIRS to understand the cortical effects of nGVS. Since it is relatively inexpensive, portable, and less prone to motion artifacts than other neuroimaging technology, fNIRS may provide insight into how nGVS affects cortical activity during a variety of natural motor and cognitive behaviors. For instance, it may be possible to determine changes in cortical HbO/HbR concentrations from nGVS in relation to performance changes during standing, walking, or spatial navigational tasks. Additional research focusing on other brain regions, different nGVS parameters, and test-retest reliability is recommended.

## Figures and Tables

**Table 1:** Region of Interest HbO and HbR average concentration differences between nGVS and sham stimulation.

HbO					
ROI	t	df	*p*-value	Conc. Diff. (10^−8^ mol)	95% CI (10^−8^ mol)
All Ch. L	0.14	11	0.887	0.75	−10.64, 12.14
All Ch. R	−0.23	11	0.822	−1.28	−13.56, 11.00
BA40 L	0.05	11	0.960	0.26	−10.64, 11.16
BA40 R	−0.54	11	0.602	−2.72	−13.89, 8.44
BA39 L	0.23	11	0.825	1.24	−10.84, 13.33
BA39 R	0.02	11	0.980	0.16	−13.50, 13.82
**HbR**					
ROI	t	df	*p*-value	Conc. Diff. (10^−8^ mol)	95% CI (10^−8^ mol)
**All Ch. L**	**2.33**	**11**	0.040 *	**2.16**	**0.12, 4.20**
All Ch. R	2.05	11	0.065	1.70	−0.12, 3.52
**BA40 L**	**2.46**	**11**	0.032 *	**2.84**	**0.30, 5.39**
BA40 R	1.94	11	0.079	2.23	−0.30, 4.77
BA39 L	1.69	11	0.118	1.48	−0.44, 3.40
BA39 R	1.12	11	0.285	1.16	−1.12, 3.44

Ch: Channels, CI: Confidence Interval, Conc: Concentration, Diff: Difference, df = degrees of freedom, L: Left, R: Right, ROI: Region of Interest. Significant results are bolded (* *p* ≤ 0.05).

**Table 2:** Per-channel HbR average concentration differences between nGVS and sham stimulation. Channels are ordered according to their *p* values.

Channel	Hemisphere	t	df	*p*-Value	Conc. Diff. (10^−8^ mol)	95% CI (10^−8^ mol)
**1**	**L**	**2.41**	**11**	0.035 *	**2.08**	**0.18, 3.99**
**7**	**R**	**2.24**	**11**	0.047 *	**2.31**	**0.04, 4.59**
2	L	2.00	11	0.071	4.35	−0.45, 9.14
3	L	1.99	11	0.073	2.21	−0.24, 4.66
10	R	1.76	11	0.106	1.43	−0.35, 3.22
5	L	1.76	11	0.107	2.09	−0.53, 4.71
9	R	1.73	11	0.111	1.96	−0.53, 4.44
8	R	1.35	11	0.204	3.18	−2.00, 8.36
6	L	1.02	11	0.332	1.46	−1.71, 4.63
4	L	0.90	11	0.386	0.76	−1.09, 2.60
11	R	0.81	11	0.436	1.21	−2.08, 4.49
12	R	0.05	11	0.965	0.10	−4.96, 5.16

CI: Confidence Interval, Conc: Concentration, Diff: Difference, df = degrees of freedom, L: Left, R: Right. Significant results are bolded (* *p* ≤ 0.05).

**Table 3:** Adverse effects post-test questionnaire results.

	Scale: 1 (None)–5 (Very Strong)				
	1	2	3	4	5
Pain	92%	8%	0%	0%	0%
Tingling	75%	25%	0%	0%	0%
Itching	92%	8%	0%	0%	0%
Burning	75%	25%	0%	0%	0%
Dizziness	100%	0%	0%	0%	0%
Fatigue	83%	17%	0%	0%	0%
Nervousness	92%	8%	0%	0%	0%
Difficulty in concentration	67%	33%	0%	0%	0%
Headache	100%	0%	0%	0%	0%
Unpleasantness	100%	0%	0%	0%	0%
Metallic Taste	100%	0%	0%	0%	0%
Visual Sensation	100%	0%	0%	0%	0%

## Data Availability

The datasets used and/or analyzed during the current study are available from the corresponding author on reasonable request.
